# Alternative inks for arbuscular mycorrhizal root staining

**DOI:** 10.1099/acmi.0.000618.v4

**Published:** 2023-08-31

**Authors:** Thomas I. Wilkes

**Affiliations:** ^1^​ School of Water, Energy and Environment, Cranfield University, Cranfield, Bedfordshire, MK43 0AL, UK

**Keywords:** arbuscular mycorrhizal fungi, ink staining, Parker ink, root tissue, Sheaffer ink

## Abstract

Alternative methods for arbuscular mycorrhizal (AM) fungal colonized root staining have recently gained more attention for the reduction of hazard exposure to the user. Sheaffer blue ink has been employed for such an identification and quantification, having shown an increased degree of image clarity. However, sourcing Sheaffer blue ink is becoming problematic, leading to the need to find alternative inks that are readily available. Parker ink is a well-known brand, providing comparable colour options to Sheaffer. Two Parker inks, blue and washable blue, were employed alongside Sheaffer blue for comparative AM fungal colonized root staining. From quantified AM fungal vesicles and arbuscles, along with the degree of stained image clarity under microscopy, none of the inks utilized for this comparison produce a significantly (*P*=0.97) different AM fungal quantification or change in image clarity. Therefore, the results of the present communication suggest that Parker blue and washable blue inks are alternative ink stains for the viewing and quantification of AM fungi in host cortical root tissues.

## Data Summary

All data pertaining to the present findings are contained within the manuscript.

## Introduction

There have been many developments in host root staining for the identification and quantification of arbuscular mycorrhizal (AM) fungi [1–4]. Sheaffer blue ink has been shown to be capable of staining AM fungi, with structures easily identifiable [5]. However, the commercial availability of Sheaffer blue ink is becoming limited, with the ink difficult to source. Therefore, a need arises for the identification of a potential substitute ink that has a comparable ability to stain AM fungal structures for easy identification. Blue inks are typically better suited for AM fungal root staining protocols for the ease of identifying differences between structures as well as atypical structures that could easily be misidentified under other staining procedures [3, 4]. Therefore, the present short communication aims to identify a potential substitute for Sheaffer blue ink in AM fungal colonized plant root staining, further utilizing commercially available blue inks.

## Methods

Zulu variety winter wheat (*Triticum aestivum*) (*n*=15) was grown under controlled conditions for 4 weeks (15 °C, 37 % relative humidity, 15 260 lux). Root staining was performed in accordance with Wilkes *et al*. [4] with Sheaffer blue ink substituted for Parker washable blue and Parker standard blue inks. Furthermore, the inclusion of formaldehyde in the plant fixative solution in Wilkes *et al*. [4] was not included in any solutions for the present samples.

Statistical analysis was performed using a single factor analysis of variance (ANOVA) and post-hoc *t*-testing utilizing R statistical software version 4.2.1. (Hamilton, ON, Canada).

## Results

**Fig. 1.**
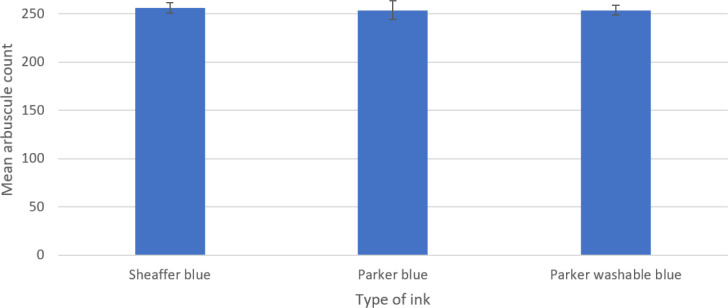
Mean (*n*=105 overall) arbuscular count of stained Zulu variety wheat 1 cm root sections stained with three different blue inks. Error bars constructed from standard error of the mean (sem).

**Fig. 2.**
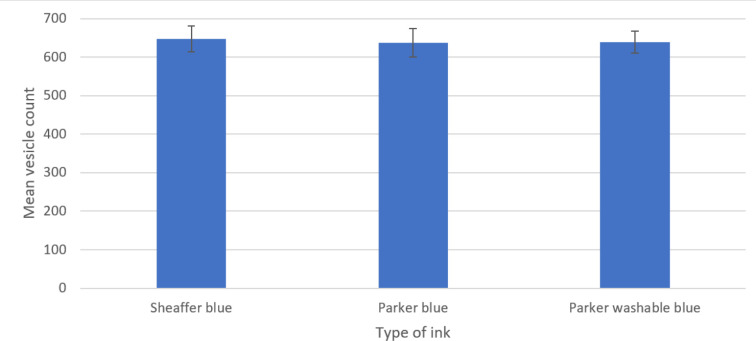
Mean (*n*=105 overall) vesicle count of stained Zulu variety wheat 1 cm root sections stained with three different blue inks. Error bars constructed from sem.

Single-factor ANOVA was able to show no significant difference between any blue inks used [*P*=0.97, degrees of freedom (df): 104, 2, f value: 0.03, f critical: 3.09] for both arbuscular (Fig. 1) and vesicular counts (Fig. 2). Post-hoc *t*-testing was not required, as no further significance could be determined.

## Discussion

**Fig. 3.**
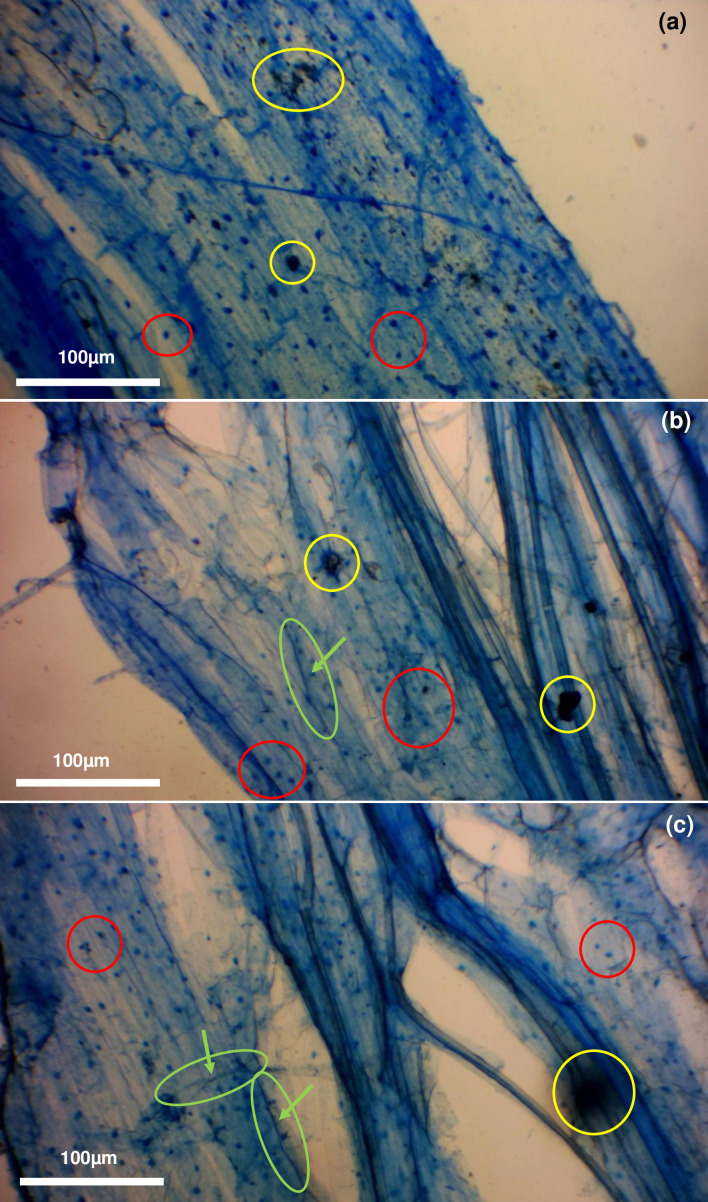
Stained Zulu variety winter wheat with (**a**) Sheaffer blue, (**b**) Parker blue and (**c**) Parker washable blue ink at 40× magnification under an Apex microscope taken with a Bresser HD microscope camera. Yellow circle, debris; red circle, vesicles; green circle, intraradical hyphae.

**Fig. 4.**
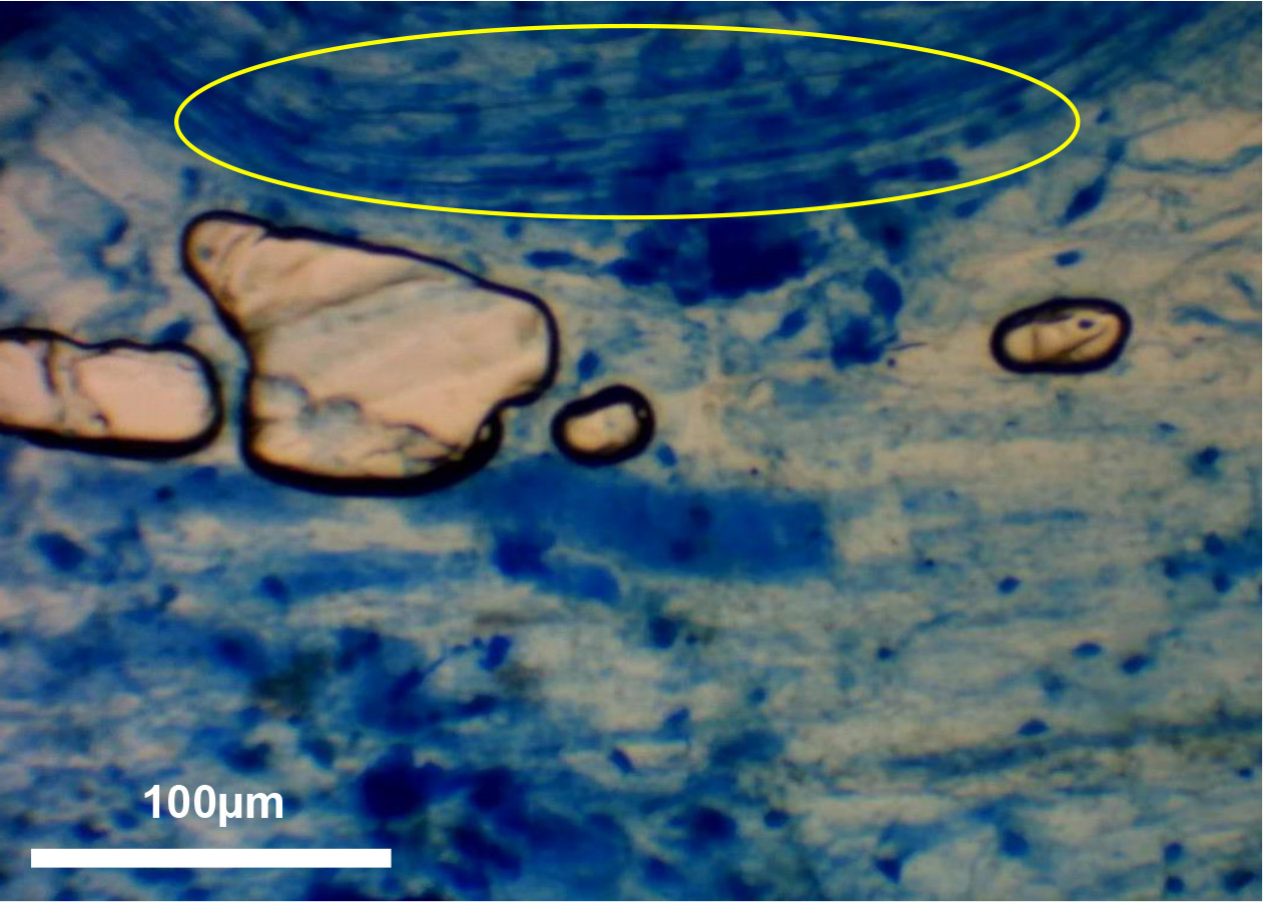
Clusters of arbuscules (yellow circle) in Zulu variety wheat as seen under an Apex microscope at 40× magnification stained with Parker washable blue ink. Image taken using a Bresser HD microscope camera.

**Fig. 5.**
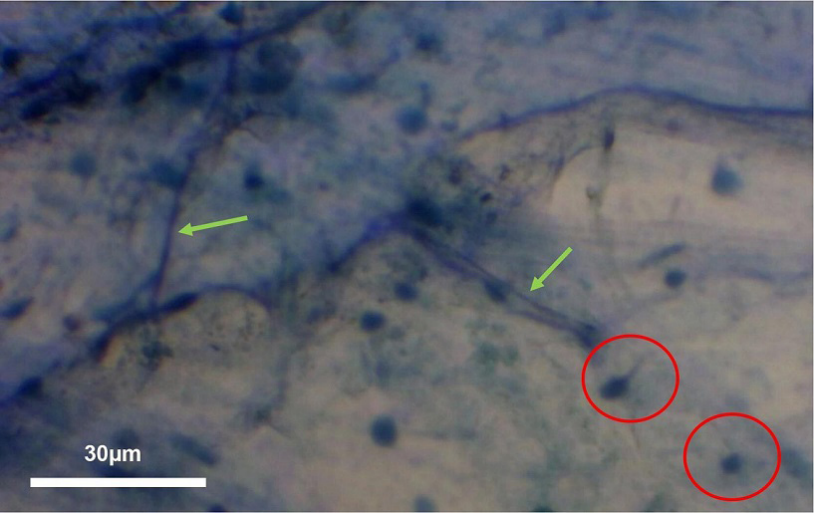
Intraradical hyphae connected to a root cortical vesicle (red circle) and stained intraradical hyphae (green arrow) in Zulu variety wheat observed under an Apex microscope at 100× magnification using Parker blue ink. Imaged taken using a Bresser HD microscope camera.

The present short communication has been able to provide indications that there is no discernible difference in AM fungal quantification between Sheaffer blue, Parker blue and Parker washable blue inks. Furthermore, as presented in Figs 3–5, there is little difference in the clarity of viewing and overall ability to quantify AM fungal root cortical structures between the three inks, allowing easy interpretation of stained tissues following the ink staining protocols developed by Hewitt *et al*. [6], Wilkes *et al*. [4] and Kowal *et al*. [5]. Yon *et al*. [7], however, did not follow the sample preparation protocols as described by Hewitt *et al*. [6], Wilkes *et al*. [4], or Kowal *et al*. [5], whilst using Parker blue and Parker washable blue inks. Micrograph images presented by Yon *et al*. [7] do not present discernible identified AM fungal structures. This is likely due to the drying of root tissues before staining, damaging the delicate AM fungal structures [4, 8]. As the present communication has been able to demonstrate, Parker blue and Parker washable blue inks are able to stain AM fungal structures. This highlights the importance of sample preparation. It is worth noting that the interpretation of Sheaffer blue-stained root tissues has been mistakenly assumed to be stained plant cell components [9]. As shown by Wilkes [8] and Wilkes and Warner [10], Sheaffer blue was not able to stain any cellular components in wheat samples grown under aseptic conditions, i.e. in the absence of AM fungi. This was further shown by micrographs presented by Kowal *et al*. [5].

## Conclusion

From AM fungal structures quantified and the clarity of micrograph images, no difference between ink brands was detectable. Therefore, the present communication can conclude Parker blue and washable blue inks are equally effective for quantifying and viewing root cortical AM fungal structures for the assessment of AM fungal–host symbiosis.
